# Examining the Role of Socioeconomic Status and Maternal Sensitivity in Predicting Functional Brain Network Connectivity in 5-Month-Old Infants

**DOI:** 10.3389/fnins.2022.892482

**Published:** 2022-06-10

**Authors:** Johanna R. Chajes, Jessica A. Stern, Caroline M. Kelsey, Tobias Grossmann

**Affiliations:** ^1^Department of Psychology, University of Virginia, Charlottesville, VA, United States; ^2^Division of Developmental Medicine, Department of Pediatrics, Boston Children’s Hospital, Boston, MA, United States; ^3^Department of Pediatrics, Harvard Medical School, Boston, MA, United States

**Keywords:** infancy, socioeconomic status, maternal sensitivity, functional connectivity, functional near-infrared spectroscopy

## Abstract

Infancy is a sensitive period of human brain development that is plastically shaped by environmental factors. Both proximal factors, such as sensitive parenting, and distal factors, such as socioeconomic status (SES), are known predictors of individual differences in structural and functional brain systems across the lifespan, yet it is unclear how these familial and contextual factors work together to shape functional brain development during infancy, particularly during the first months of life. In the current study, we examined pre-registered hypotheses regarding the interplay between these factors to assess how maternal sensitivity, within the broader context of socioeconomic variation, relates to the development of functional connectivity in long-range cortical brain networks. Specifically, we measured resting-state functional connectivity in three cortical brain networks (fronto-parietal network, default mode network, homologous-interhemispheric connectivity) using functional near-infrared spectroscopy (fNIRS), and examined the associations between maternal sensitivity, SES, and functional connectivity in a sample of 5-month-old infants and their mothers (*N* = 50 dyads). Results showed that all three networks were detectable during a passive viewing task, and that maternal sensitivity was positively associated with functional connectivity in the default mode network, such that infants with more sensitive mothers exhibited enhanced functional connectivity in this network. Contrary to hypotheses, we did not observe any associations of SES with functional connectivity in the brain networks assessed in this study. This suggests that at 5 months of age, maternal sensitivity is an important proximal environmental factor associated with individual differences in functional connectivity in a long-range cortical brain network implicated in a host of emotional and social-cognitive brain processes.

## Introduction

Infancy is a sensitive period in human brain development (Johnson and de Haan, 2015; Gilmore et al., 2018) marked by rapid neural growth. The brain development and organization that happens during infancy serves as the basis for future brain function and related behavioral outcomes (Tau and Peterson, 2010; Kolb and Gibb, 2011; Bornstein, 2014). The infant brain is highly plastic and changes in response to environmental inputs (Tierney and Nelson, 2009; Dawson et al., 2012; Hodel, 2018; Rebello et al., 2018; Merz et al., 2019). These environmental factors exist within various systems or levels of proximity to an individual, as outlined in Bronfenbrenner’s bioecological framework (Bronfenbrenner, 1974; Bronfenbrenner and Ceci, 1994) and in modern neuroconstructivist views (Westermann et al., 2007). This model highlights the importance of considering how environmental factors within and across each of these systems may work together to exert influence on every aspect of child development, including the highly malleable infant brain.

One of the more distal factors thought to play an important role in brain development is socioeconomic status (SES). Broadly defined, SES is how “well off” an individual is within their society, and this often is operationalized as one’s access to material and nonmaterial resources, such as income and educational attainment (Farah, 2017), though many different conceptualizations exist in the literature (see Antonoplis, in press). Higher SES is associated with a wide variety of positive developmental outcomes, including better physical and mental health, higher academic achievement, and improved neurocognitive functioning (Adler and Newman, 2002; Bradley and Corwyn, 2002; Noble et al., 2005; Ursache and Noble, 2016; Kivimäki et al., 2020). Increasingly, SES is not only considered as a covariate that should be accounted for but rather viewed as a key component in studying the brain’s structure and function early in life (for a review, see Noble and Giebler, 2020; Olson et al., 2021) and into adulthood (Chan et al., 2018). Growing up in a low-SES environment has repeatedly been shown to be a risk factor for altered brain development, predicting decreased gray and white matter volume and less mature functional brain networks, possibly due to an accelerated pace of functional maturation in response to stress (Hanson et al., 2013; Tooley et al., 2021). SES is also associated with functional connectivity levels for several brain networks, including the default mode network, sensorimotor network, and brain circuits connecting the prefrontal cortex to deeper brain structures like the striatum and amygdala, within the first 6 months of life (Gao et al., 2015; Turesky et al., 2019; Ramphal et al., 2020). Beyond these correlational findings, poverty reduction interventions also suggest a causal role in the associations between SES and brain development (Troller-Renfree et al., 2022). For example, when low-income families were randomly assigned to receive either large or nominal monthly cash gifts shortly after giving birth, infants in the high-cash group exhibited greater EEG power in higher-frequency bands (beta and gamma) compared to infants in the low-cash group a year later, although these differences did not survive multiple comparisons. These findings both demonstrate that this type of intervention can result in differences in infant brain activity while they also indicate that these differences may only be small in effect size.

In addition to distal environmental factors such as SES, there are other factors that shape infant brain development at a more proximal level and may thus exert a more direct influence on infants’ everyday experiences. One such factor, the quality of the mother–infant relationship, is thought to influence both brain and behavioral outcomes for the child (Parsons et al., 2010). Maternal sensitivity, more specifically, is among the most robust predictors of positive child outcomes such as self-regulation and social adjustment (Cassidy and Shaver, 2016; Deans, 2020). Observational measures of maternal sensitivity are considered a gold-standard way to assess caregiving quality. Two widely used dimensions are *maternal sensitivity vs. insensitivity*—prompt and appropriate responsiveness to the infant’s signals (e.g., timely soothing of crying/distress), and *maternal cooperation vs. interference—*support for the infant’s autonomy, facilitation of exploration, and minimal interference with the infant’s ongoing activities (e.g., allowing the infant to play with a novel toy without the mother’s interruption or directives) (Ainsworth, 1969, 1979). Decades of evidence show that maternal sensitivity and cooperation (henceforth simply called sensitivity) relate to positive behavioral outcomes (for a review see Deans, 2020), as well as physical health outcomes in infancy (e.g., Stern et al., 2020). Emerging research suggests that maternal sensitivity relates to child brain structure (e.g., Perry et al., 2017; Bernier et al., 2019) and function (see Tottenham, 2018) and evidence for a causal impact of sensitivity on brain development comes from parenting interventions designed to increase maternal sensitivity (Valadez et al., 2020). Maternal sensitivity has also been shown to relate to resting state functional connectivity between cortical and subcortical brain structures during infancy (Rifkin-Graboi et al., 2015; Wang et al., 2019) and in higher-order brain networks like the fronto-parietal network, default mode network, and salience network during childhood (Dégeilh et al., 2018; Pozzi et al., 2021). However, to date there is no study which has examined associations between maternal sensitivity and functional connectivity in these higher-order brain networks during early infancy. Critically, previous work attests that these higher-order networks can be identified and its variability can be assessed in infants as early as 1 month postnatally (Kelsey et al., 2021a).

Thus, both SES and maternal sensitivity are strong predictors of future behavioral outcomes and are known to affect brain development early in human development. Previous work suggests that these two environmental factors are also associated with one another (see Bornstein et al., 2007). SES is theorized to influence children’s brain development through increased environmental stress (Noble and Giebler, 2020; Tooley et al., 2021), and a similar mechanism could explain how SES impacts maternal sensitivity (Farah, 2017; Neuhauser, 2018). Additionally, the positive effects of high maternal sensitivity could act as a protective factor against exposure to low-SES environments during early development (Luby et al., 2013), with positive parenting intervention studies in low-SES populations suggesting a potential buffering role (Brody et al., 2017). Critically, however, SES and maternal sensitivity have yet to be explored together in their effects on the development of functional connectivity during infancy, which is an important step toward understand how maternal sensitivity and SES work together in shaping the infant brain. Existing theories have proposed that parental sensitivity may serve as an explanatory factor (mediator) as well as a protective factor (moderator) of the negative outcomes associated with low SES (e.g., Bornstein and Bradley, 2002; Conger et al., 2010), yet these theories have not been put to the test early in ontogeny. The current study aims to address this gap.

Using data from a longitudinal study of mothers and infants, we examined how both distal (family SES) and proximal (maternal sensitivity) familial factors contribute to the development of long-range functional cortical networks in the infant brain during the first 6 months of life. We chose to focus on the 5-month time point specifically because our long-range functional networks of interest are known to be present at this age (Gao et al., 2009, 2017; Kelsey et al., 2021a), and previous work suggests that the infant brain may exhibit unique associations between SES and functional connectivity at this age (Gao et al., 2015). Resting-state functional connectivity among networks in the infant brain was measured by functional near-infrared spectroscopy (fNIRS). fNIRS is a safe, non-invasive, and therefore infant-friendly, optical neuroimaging technique that provides an indirect measure of surface-level cortical brain function by detecting changes in the concentration of oxygenated (and deoxygenated) hemoglobin in different areas of the cortex (for a review of this technique and how it is used in infant neuroimaging studies, see Lloyd-Fox et al., 2010; Boas et al., 2014; Mohammadi-Nejad et al., 2018; Hu et al., 2020; Yeung, 2021). We focused specifically on three, pre-defined higher-order functional brain networks, the fronto-parietal network (FPN), the default mode network (DMN), and a network of homologous-interhemispheric connections (HI), as these networks can be assessed during infancy using fNIRS (e.g., Bulgarelli et al., 2020; Kelsey et al., 2021a) and are known to develop throughout the first year of life and thus may be particularly susceptible to shaping by external factors like SES and maternal sensitivity (Gao et al., 2009, 2015; Gilmore et al., 2018; Kelsey et al., 2021a). The FPN is composed of the rostral and dorsolateral prefrontal cortex, anterior cingulate cortex, and regions of the parietal cortex, and is thought to play a role in cognitive control and executive functioning, a behavioral outcome that often exhibits SES-related differences (Merz et al., 2019; Noble and Giebler, 2020). The DMN consists of the medial prefrontal cortex, posterior cingulate cortex, precuneus, inferior parietal cortex, and lateral temporal cortex, and is involved in social cognition and internally-oriented thought (Raichle et al., 2001; Buckner and DiNicola, 2019). It is one of the first higher-order functional networks to fully develop during infancy (Gao et al., 2009) and differences in DMN connectivity have been observed in relation to both SES (e.g., Gao et al., 2015) and maternal behavior (e.g., Dégeilh et al., 2018). Lastly, the HI is made up of cross-hemispheric connections between homologous regions in the frontal, parietal, and temporal lobes. It provides a useful measure of cross-hemispheric connectivity throughout the cortex, and is thought to be related to emotion regulation, an outcome that has also been tied to differences in maternal sensitivity (e.g., Deans, 2020). We compared the level of connectivity in these three functional networks to a (non-functional) control network (CN) made up of connections between the left frontal and right temporal cortex and the right frontal and left temporal cortex. This kind of control network has been used in previous fNIRS studies (see Homae et al., 2010; Sasai et al., 2011; Kelsey et al., 2021a) and serves as a non-functional baseline measure of connectivity since these brain regions are not known to have any functional associations. It is important to acknowledge that while some of these functional networks do contain subcortical regions (i.e., anterior and posterior cingulate cortex, precuneus) due to the limitations of fNIRS which can only measure about 3 cm deep into the cortex, we will be focusing only on the outermost portions of each network in accordance with other similar studies of infant functional brain connectivity (e.g., Bulgarelli et al., 2020; Kelsey et al., 2021a).

As an initial step, we aim to establish whether our three pre-defined functional networks exhibit greater functional connectivity than a non-functional control network at this age. Next, we address three main research questions relating to the associations between SES, maternal sensitivity, and infant functional brain connectivity: (1) How does SES, measured as annual household income and maternal education, relate to the development of functional brain connectivity during infancy; (2) Do more proximal factors like maternal sensitivity mediate the association between SES and functional brain development; and (3) Does maternal sensitivity buffer against potential detrimental effects of low SES on brain development?

In our preliminary analyses, we hypothesized that at 5 months of age, resting-state functional connectivity in these three functional networks will be significantly greater than in the CN. Regarding the central research questions, we first predicted that SES will be associated with individual differences in network connectivity in the three functional networks compared to the CN (Hypothesis 1), but given a lack of previous findings looking at the impact of SES on functional connectivity in relation to a non-functional control network, we did not specify a particular direction for this hypothesis. Second, we hypothesized that maternal sensitivity, measured during parent-child interactions at 5 months, will mediate the effect of SES on connectivity in the FPN, DMN, and HI (Hypothesis 2). Third, we predicted that maternal sensitivity may act as a moderator, such that high maternal sensitivity will buffer against the effect of SES on network connectivity in the three functional networks (Hypothesis 3). All hypotheses were pre-registered (osf.io/hcg42).

## Materials and Methods

### Participants

Participants are part of a larger longitudinal study of social and emotional development and 121 mother-infant dyads were first recruited from a local hospital when the infants were newborns (for details see Kelsey et al., 2021a,b). In order to enroll, participants had to be born at term, with normal birth weight (>2,500 g), and did not have any hearing or visual impairments. When infants were 1 month of age (*M* age = 31.8 days; *SD* = 26.4 days; range = 9–141 days; *N* = 121, *n* = 72 male sex assigned at birth), mothers completed a series of questionnaires which included questions about socioeconomic status. Of the original 121 dyads, 109 families returned to the lab when the infants were 5 months (*M* age = 5.2 months; *SD* = 0.68 months; range = 4–7 months; *N* = 109, *n* = 69 male sex assigned at birth) and the infants participated in an fNIRS recording session and a free-play session. Of the 109 dyads that completed the 5-month visit, 50 were included in the current analytic sample (*M* age = 5.2 months; *SD* = 0.67 months; range = 4–7 months; *N* = 50, *n* = 31 male sex assigned at birth) (for dropout or data exclusion, see details below). Families who were included in the current analytic sample did not significantly differ from those who completed the 5-month visit but were not included on any sociodemographic characteristics except number of children in the household, with excluded families having more children (*M* = 2.30; *SD* = 1.03, *N* = 59) on average than included families (*M* = 1.86; *SD* = 1.08, *N* = 50, *p* = 0.027). See Table 1 for sample sociodemographic characteristics. Parents provided informed consent on behalf of their child in accordance with the Declaration of Helsinki. All procedures were approved by the authors’ institutional review board, and participants received monetary compensation for participation.

**TABLE 1:** Sociodemographic characteristics of included participants.

Sociodemographic characteristic	Mean (*M*) or Count (*n*)	*SD* or %
Infant age in months at 5-month time point, *M*	5.20	0.67
**Infant sex assigned at birth, *n***		
Male	31	62%
Female	19	38%
**Infant race, *n***		
Black	1	2%
White	37	74%
South Asian	1	2%
Multiracial	9	18%
Other/self-specify	2	4%
**Household income, *n***		
Less than $15,000	5	10%
15,001 to $30,000	5	10%
30,001 to $45,000	6	12%
45,001 to $60,000	7	14%
60,001 to $75,000	3	6%
75,001 to $90,000	4	8%
90,001 to $110,000	10	20%
110,001 to $125,000	2	4%
125,001 to $175,000	6	12%
175,001 to $225,000	1	2%
More than $225,000	1	2%
**Maternal education, *n***		
Some high school	1	2%
High school diploma/GED	6	12%
Some college/Associate degree	10	20%
Bachelor’s degree	17	34%
Graduate degree (e.g., Masters or Ph.D.)	16	32%
Number of children, *M*	1.86	1.08
Maternal sensitivity score (1–9), *M*	5.99	1.87
Maternal cooperation score (1–9), *M*	5.49	2.15

*N = 50.*

Fifty-nine additional infants returned for the 5-month testing session but were excluded from the present analyses for the following reasons: *n* = 35 were excluded for having more than 50% of fNIRS channels excluded during preprocessing (see below for details); *n* = 16 were excluded due to technical errors; *n* = 7 were excluded because they did not have at least 100 s of continuous fNIRS data with non-disruptive behaviors (see below); *n* = 1 was excluded because of inaccurate placement of the fNIRS cap (more than 1 cm deviation from proper cap placement).

### Procedure

#### Socioeconomic Status

Socioeconomic status (SES) was assessed using a parent questionnaire at the 1-month time point. Mothers filled out the questionnaire online using Qualtrics survey platform prior to their first, newborn appointment. SES was determined based on reported annual household income (“In the household where this child primarily lives, what is the total annual household income?”) and maternal education (“Education”) (see Table 1 for categorical response options).

#### Maternal Sensitivity

Maternal sensitivity was assessed from video recordings of mother-infant interactions during the 5-month time point. Mothers were invited into a laboratory playroom for a 5-min free-play session. Infants were placed on their backs on a blanket in the center of the room, and an experimenter instructed mothers to “play with your child as you normally would” for 5 mins, following procedures from Grossmann et al. (2018). Two cameras provided simultaneous recordings of the session: one camera captured the mother’s face and body, and one captured the infant. Each dyad was provided with a standardized set of objects (toys and a book). Mothers and infants could freely choose which object(s) they engaged with, if any.

Videos of mother-infant interactions were scored by a team of four trained coders using two scales from Ainsworth’s (1969) gold-standard Sensitivity Scales: *sensitivity vs. insensitivity to infant signals* (i.e., maternal sensitivity) and *cooperation vs. interference with infant’s ongoing activity* (i.e., maternal cooperation). Ratings range from 1 to 9 for each scale, with higher scores reflecting more sensitive or cooperative behavior (sensitivity: 1 = highly insensitive, 5 = inconsistently sensitive, 9 = highly sensitive; cooperation: 1 = highly interfering, 5 = mildly interfering, 9 = conspicuously cooperative). One hundred percent of videos were double-coded for reliability by two independent raters, and discrepancies were resolved via conferencing. Interrater reliability was good for sensitivity (Krippendorff’s alpha = 0.70) and cooperation (K-alpha = 0.75). Because scores for maternal sensitivity and cooperation are typically strongly correlated (*r* ∼ 0.80), they were averaged to create a *maternal sensitivity composite* for all main analyses, following previous work (e.g., Stern et al., 2020).

#### Infant Functional Connectivity

Infants’ resting-state functional connectivity was assessed using fNIRS at the 5-month time point.

##### Functional Near-Infrared Spectroscopy Data Collection

Infants were seated on their parents’ lap and sat approximately 60 cm from the screen (23-inch monitor). The infants wore an fNIRS fabric cap (EasyCap, Germany) which was secured in place using infant overalls and outside netting. Stimuli were presented using the Presentation software package (Neurobehavioral Systems, United States). The video stimulus was played for a total of 7 mins while fNIRS data were being recorded. Parents were asked to remain quiet throughout the fNIRS recording session. Sessions were video-recorded to allow offline coding of infants’ behavior and cap placement.

##### Stimuli

The non-social stimulus was created by selecting non-social video clips (e.g., toys, fruits, and everyday objects) from a popular infant video (Baby Einstein - Kids2 Inc.). The images were accompanied by classical music (Lordier et al., 2019). The video was segmented into 30-s intervals and the order of presentation was randomized for each infant. This paradigm has been successfully used in previous work (Kelsey et al., 2021a,b).

##### Data Acquisition

**FIGURE 1:**
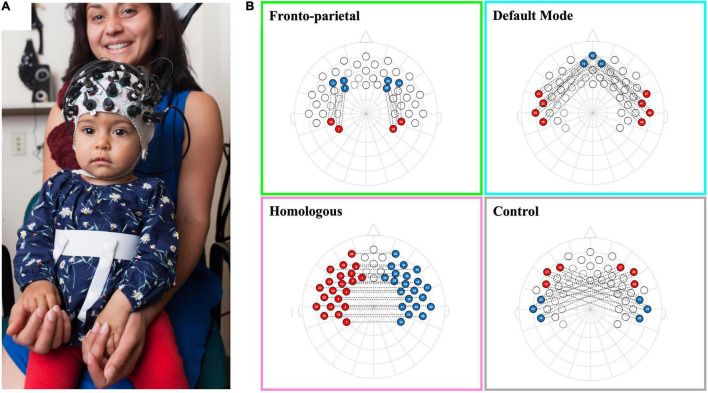
Schematic of infant network configurations measured using fNIRS. Panel **(A)** shows a infant wearing a functional near-infrared spectroscopy (fNIRS) cap with our 10–20 system optode array. Panel **(B)** shows the configurations for each of the network patterns in a 2-dimentional 10–20 system layout. Each network consists of the average of the correlations between each red-blue channel pair that is connected by a dotted line.

Infants’ fNIRS data were recorded using a NIRx Nirscout system and NirStar acquisition software. The fNIRS system used has 49 channels (approximately 2 cm source-detector distance) with extensive coverage over the frontal and temporal-parietal regions (see Altvater-Mackensen and Grossmann, 2016; Grossmann et al., 2018; Kelsey et al., 2019b; Krol et al., 2019b for infant work using the identical channel positioning/layout; see Figure 1A). The system emits two wavelengths of light, 760 and 850 nm, and captures both oxygenated hemoglobin (oxyHb) and deoxygenated hemoglobin (deoxyHb). The diodes have a power of 25 mW/wavelength and data were recorded at a preset default sampling rate of 3.91 Hz.

##### Behavioral Coding

Infants’ behavior during the fNIRS recording session was coded by a trained research assistant using video recordings of the experimental session and following a previous behavior coding scheme used in Kelsey et al. (2021a). Specifically, the coder flagged time points for removal where the infants were crying, infants were social referencing (looking to the parent or experimenter), or parents were talking. To assess the reliability of the behavioral coding done by the primary coder, and additional trained research assistant coded a selected subsample of infants (28.4%, *n* = 31). Interrater reliability was excellent (Intraclass correlation coefficient = 0.92 [95% CI, 0.83–0.96]). As part of our pre-registered processing plan and based on previous studies, infants needed a minimum of 100 s of disruption-free data to be included (Bulgarelli et al., 2019). On average, infants included in the final sample contributed 365.68 s of data (*SD* = 105.84 s; range = 162–420 s; see Supplementary Figure 1).

##### Pre-registered Functional Near-Infrared Spectroscopy Processing Plan

The fNIRS data were analyzed using custom Matlab scripts and Homer2 following the plan outlined in the pre-registration and in accordance by guidelines outlined by Powell (2020). Specifically, (1) raw intensity data were converted to optical density units, (2) channels with mean intensities outside the system recommended values (enPrunechannels: dmin = 10^–2^, dmax = 10^9^) were removed, (3) flexible targeted Principal Component Analysis (Yücel et al., 2014) with up to three iterations (tMotion = 1.0, tMask = 1.0, Std Thresh = 100, Amp Thresh = 0.1, tpcaFilter = 0.97) were used to correct for motion artifacts (see Blanco et al., 2021; Baek et al., 2022; for other examples of motion correction used in infant fNIRS functional connectivity analyses), (4) the corrected data were band-passed filtered (0.01–0.08 Hz) (Bulgarelli et al., 2019; Kelsey et al., 2021a), (5) data were converted into oxygenated and deoxygenated hemoglobin concentration change values using a modified Beer Lambert Law assuming a partial path length factor of 5 commonly used with infants of a similar age (hmrOD2Conc) (Pirazzoli et al., 2019; Porto et al., 2020). For each infant, a 49 by 49 correlation matrix was created corresponding to all of the relations between all of the channels measured. Correlation values were standardized using a Fisher Z-transformation.

Networks of interest were pre-registered and created from an average template by selecting channels that corresponded to specific regions of interest (see Kelsey et al., 2021a for previous manuscripts using this approach). Brain areas were named in accordance to anatomical mappings of the 10–20 system in similar age infants (see Kabdebon et al., 2014), and were confirmed at the group level based on the LONI probabilistic brain atlas (LPBA) using photon propagation simulation with realistic, age-appropriate (6.0 months) head models (see devFOLD toolbox for more details; Fu and Richards, 2021). We created four networks: (1) The front-parietal network (FPN), created by averaging the left and right hemisphere correlations between three channels in the dorsolateral prefrontal cortex [corresponding with the F3, F4, F5, and F6 electrodes (10–20 system), or the middle frontal gyrus (LPBA)] and two channels in the parietal area [corresponding with CP3 and CP4 electrodes (10–20 system), or the supramarginal and postcentral gyri (LPBA)]; (2) The default mode network (DMN) was created by averaging the left and right hemisphere correlations between three channels in the medial prefrontal cortex [corresponding with the Fpz electrode (10–20 system), or the superior and middle frontal gyri (LPBA)] and four channels in the lateral temporal cortex [corresponding with FT7, T7, FT8, and T8 electrodes (10–20 system), or the superior, middle, and inferior temporal gyri (LPBA)]; (3) The network of homologous-interhemispheric connections (HI) was created by averaging the correlations between 21 channels in the left hemisphere (including frontal, temporal, and parietal cortical regions) with their corresponding (homologous) channels in the right hemisphere [all frontal, parietal, and temporal gyri (LPBA)]; and (4) The (non-functional) control network (CN) was created by averaging all correlations between three channels in the left or right frontal areas [corresponding with the F7 or F8 electrodes (10–20 system), or the inferior frontal gyrus (LPBA)] with three channels in the right or left temporal areas [corresponding with the T8 or T7 electrodes (10–20 system), or the superior and middle temporal gyri (LPBA)], respectively. See Figure 1B for a schematic of network configurations.

We conducted all analyses for both oxyHb and deoxyHb (for deoxyHb results see Supplementary Material). We examined both chromophores because they are physiologically linked (i.e., as oxyHb goes up, deoxyHb goes down), and therefore we would expect to observe similar patterns in functional connectivity for both (Lloyd-Fox et al., 2010; Bulgarelli et al., 2020; Kelsey et al., 2021a). All analyses were carried out using SPSS v28 (SPSS Inc., Chicago, IL), and no statistical outliers (values that were more than 3 SDs above or below the mean) were found in our included sample (*N* = 50).

## Results

### Preliminary Analyses

All data analyses were pre-registered. First, a series of Spearman’s rho correlations was used to identify significant associations between the main variables of interests (household income, maternal education, maternal sensitivity, maternal cooperation, FPN connectivity, DMN connectivity, HI connectivity, CN connectivity) and potential covariates (FC seconds, infant sex assigned at birth, mother race, infant race, number of children) (see Table 2). As an exploratory analysis, Spearman’s rho correlations between the variables of interest and the number of usable fNIRS channels were also examined (see Supplementary Material). Spearman’s rho correlations were used since several of our potential covariates were ordinal variables (infant sex, mother race, infant race, and number of children). Only covariates that were significantly associated with a main variable of interest were included as covariates in subsequent analyses.

**TABLE 2:** Correlations among study variables.

Variable	1	2	3	4	5	6	7	8	9	10	11	12	13
(1) Household income	–												
(2) Maternal education	0.575**	–											
(3) Maternal sensitivity vs. insensitivity scale	0.079	0.023	–										
(4) Maternal cooperation vs. interference scale	–0.030	–0.177	0.841**	–									
(5) FPN connectivity	–0.050	0.050	0.009	0.003	–								
(6) DMN connectivity	0.112	0.003	0.244	0.347*	0.155	–							
(7) HI connectivity	0.111	–0.133	–0.181	–0.086	0.394**	0.276	–						
(8) CN connectivity	0.197	0.188	–0.055	–0.037	0.167	0.359*	0.430**	–					
(9) FC seconds	0.010	–0.039	–0.272	–0.176	0.097	0.120	0.058	0.130	–				
(10) Infant sex assigned at birth	–0.161	–0.203	0.250	0.196	–0.050	0.198	–0.021	0.016	–0.023	–			
(11) Mother race	–0.058	–0.027	0.213	0.126	–0.150	0.050	–0.246	0.058	–0.038	0.040	–		
(12) Infant race	–0.183	–0.101	0.079	–0.056	–0.177	–0.065	–0.128	0.033	–0.175	0.194	0.549**	–	
(13) Number of children	–0.240	−0.326*	–0.268	–0.275	–0.078	–0.029	0.163	–0.134	0.058	0.039	–0.089	–0.021	–

*FPN, fronto-parietal network; DMN, default mode network; HI, homologous-interhemispheric connections; CN, control network; FC seconds, number of seconds for which functional connectivity data were available for each participant. Dichotomous variables include infant sex assigned at birth (1 = Male, 2 = Female), and mother and infant race (1 = White, 2 = Not White). N = 50.*

*p < 0.05, **p < 0.01, two-tailed.

Of all the network connectivity measures, only the DMN was significantly associated with one of the other non-network variables of interest [maternal cooperation: *r*_(48)_ = 0.347, *p* = 0.013]. Of all the potential covariates, only number of children was significantly associated with maternal education [*r*_(47)_ = −0.326, *p* = 0.022]; no other covariates were significantly associated with any of the SES, maternal behavior, or functional connectivity variables of interest (all *p*s > 0.056). Notably, the amount of usable functional connectivity data collected during the fNIRS testing session (“FC seconds”) was not significantly associated with any of the functional connectivity measures (all *p*s > 0.368), indicating that the amount of data included for each participant was not related to network connectivity.

Given the large positive correlation between maternal sensitivity and maternal cooperation [*r*_(48)_ = 0.841, *p* < 0.001], these variables were averaged together to create a maternal sensitivity composite score, which was used in all main analyses involving maternal behavior (in line with previous work; Stern et al., 2020). Similarly, there was a large positive correlation between household income and maternal education [*r*_(48)_ = 0.575, *p* < 0.001], so each variable was transformed into a Z-score and then summed to create an SES composite score, following our pre-registered criteria. Results from the unadjusted models are reported below, and the adjusted models controlling for number of children are reported in the Supplementary Material; all meaningful differences between the adjusted and unadjusted models have been included below.

**FIGURE 2:**
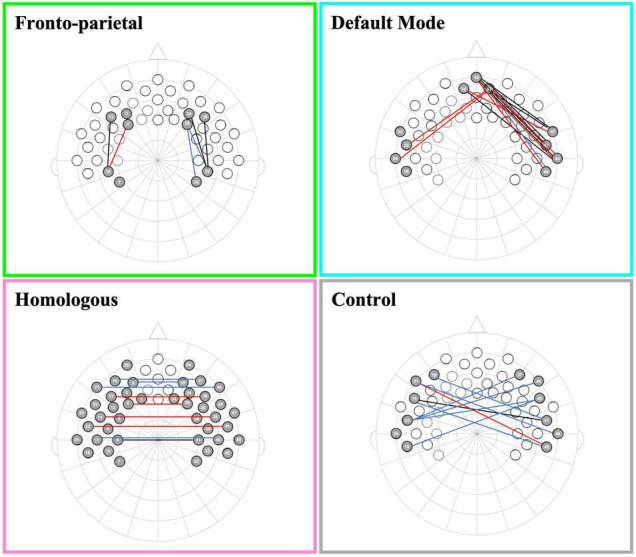
Channel pair connectivity by network. This figure shows the channel pairs with functional connections that were significantly different from zero for each network. Connections in red, blue, and black represent significant changes between the channels for oxyHb, deoxyHb, and both oxy and deoxyHb, respectively.

**FIGURE 3:**
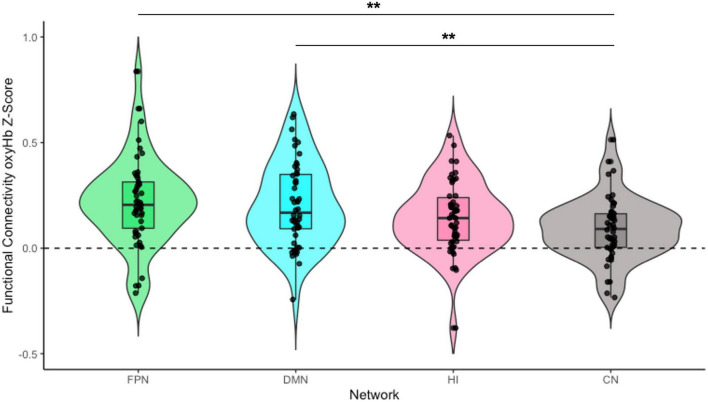
Infant functional connectivity by network at 5 months. This figure shows the average levels of functional connectivity (oxyHb) for each network. The boxplot horizontal lines from bottom to top reflect values for the lower quartile, median, and upper quartile, respectively. FPN, fronto-parietal network; DMN, default mode network; HI, homologous-interhemispheric connections; CN, control network. *N* = 50. **p < 0.01.

To address our preliminary hypotheses, we assessed functional connectivity levels within and across all four networks. A series of one-sample *t*-tests was conducted to assess whether Fisher-transformed correlations between individual channels within each pre-defined network differed from zero. As shown in Figure 2, this analysis identified significant functional connectivity between individual channels within the pre-defined networks of interest (see Supplementary Table 1). Another series of one-sample *t*-tests was conducted to assess network-level connectivity by combining across all channels of interest. All four networks were greater than zero [FPN: *t*_(48)_ = 7.44, *p* < 0.001, *d* = 0.21; DMN: *t*_(49)_ = 7.49, *p* < 0.001, *d* = 0.20; HI: *t*_(49)_ = 6.11, *p* < 0.001, *d* = 0.17; CN: *t*_(49)_ = 4.31, *p* < 0.001, *d* = 0.15; see Figure 3]. Finally, we examined whether there were differences in connectivity between the right and left hemispheres of both the FPN and DMN in an exploratory analysis (see Supplementary Material).

To examine differences in overall connectivity levels across the four networks, an omnibus repeated measures ANOVA (rmANOVA) was conducted with network type (FPN, DMN, HI, and CN) as a within-subject factor. The analysis revealed a significant within-subject effect across network types [*F*_(3, 144)_ = 6.76, *p* < 0.001, η^2^_partial_ = 0.12]. Post hoc analysis with a Bonferroni adjustment revealed that FPN connectivity (*M* = 0.22, *SD* = 0.21) was significantly greater than CN connectivity (*M* = 0.10, *SD* = 0.15) {pairwise comparison (*M_*FPN*_* − *M*_*CN*_ = 0.12 [95% CI, 0.03–0.21], *p* = 0.002)}. DMN connectivity (*M* = 0.21, *SD* = 0.20) was also significantly greater than CN connectivity {pairwise comparison (*M_*DMN*_* − *M*_*CN*_ = 0.11 [95% CI, 0.03–0.19], *p* = 0.003)}. However, there was no significant difference between HI connectivity (*M* = 0.15, *SD* = 0.17) and CN connectivity {pairwise comparison (*M_*HI*_* − *M*_*CN*_ = 0.06 [95% CI, −0.004 to 0.13], *p* = 0.079)}. There were no significant differences in connectivity among the FPN, DMN, and HI (all *p*s > 0.199; see Figure 3).

### Socioeconomic Status and Functional Connectivity

To assess how differences in family socioeconomic status were associated with infant functional connectivity, separate rmANOVAs were conducted for each functional network of interest (FPN, DMN, and HI) to compare each functional network directly with the non-functional control network (CN). For each rmANOVA, the functional network of interest and the CN were entered as within-subject factors. Additionally, in order to include SES as a between-subjects factor, a dichotomized SES score was created using a median split of the SES composite, and this score was entered as a between-subjects factor in each rmANOVA.

For each functional network, there was a significant within-subject effect of network such that connectivity was greater in the functional network than in the control network {FPN: [*F*_(1, 47)_ = 15.00, *p* < 0.001, η^2^_partial_ = 0.24]; DMN: [*F*_(1, 48)_ = 15.75, *p* < 0.001, η^2^_partial_ = 0.25]; HI: [*F*_(1, 48)_ = 5.54, *p* = 0.023, η^2^_partial_ = 0.10]}. In the HI adjusted model, there was no longer a significant within-subject effect of network [*F*_(1, 46)_ = 0.06, *p* = 0.807, η^2^_partial_ = 0.001]. Contrary to hypotheses, SES was not significantly associated with connectivity in any functional brain network, regardless of covariates or analytic approach (all *p*s > 0.180). Additionally, exploratory analyses using an alternative SES variable equal to annual household income divided by the number of children in the household yielded similar non-significant results (see Supplementary Material).

### Maternal Sensitivity as a Mediator of the Association Between Socioeconomic Status and Functional Connectivity

**FIGURE 4:**
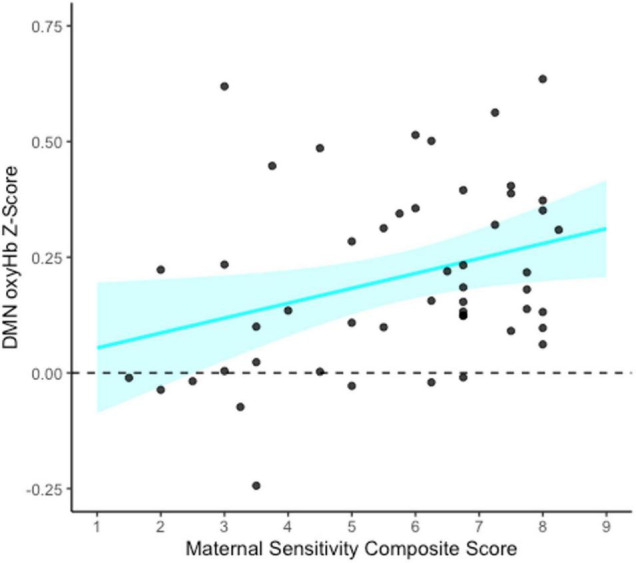
Association between maternal sensitivity composite and infant default mode network (DMN) connectivity at 5 months. This figure shows the unadjusted relation between the maternal sensitivity composite (average of maternal sensitivity vs. insensitivity and cooperation vs. interference scales) and DMN functional connectivity (oxyHb) Z-scores. The blue shaded area represents the upper and lower bounds of the mean 95% confidence interval for the raw data. *N* = 50.

To assess whether maternal sensitivity served as a mediator of a potential indirect relation between family socioeconomic status and infant functional connectivity, bootstrapped mediation models were conducted for each functional network of interest (FPN, DMN, and HI). As an exploratory analysis, a bootstrapped mediation model was also conducted for the non-functional control network (see Supplementary Material). All models were conducted using 1,000 bootstrapped samples with PROCESS v4.0 in SPSS (Hayes, 2022). Specifically, the functional network of interest was entered as the outcome variable (Y), SES composite as the predictor variable (X), and maternal sensitivity composite as the mediator (M). SES was not associated with maternal sensitivity, and thus no mediation models were significant (all *p*s > 0.889). However, maternal sensitivity composite was significantly associated with greater infant DMN connectivity specifically (*b* = 0.03, *p* = 0.025, see Figure 4).

### Maternal Sensitivity as a Moderator of the Association Between Socioeconomic Status and Functional Connectivity

To assess whether maternal sensitivity interacts with family socioeconomic status to predict infant functional connectivity, bootstrapped moderation models were conducted for each functional network of interest (FPN, DMN, and HI). As an exploratory analysis, a bootstrapped moderation model was also conducted for the non-functional control network (see Supplementary Material). All models were conducted using 1,000 bootstrapped samples with PROCESS v4.0 in SPSS (Hayes, 2022). Specifically, the functional network of interest was entered as the outcome variable (Y), SES composite as the predictor variable (X), and maternal sensitivity composite as the moderator (W). There was no significant interaction between SES and maternal sensitivity composite for any of the functional connectivity network outcome variables, and thus no moderation models were significant (all *p*s > 0.370).

## Discussion

Theories like Bronfenbrenner’s classic bioecological model (Bronfenbrenner, 1974; Bronfenbrenner and Ceci, 1994) and modern neuroconstructivist views (Westermann et al., 2007) point to the vital importance of context in shaping early development. The present study examined the unique and interactive contributions of proximal (maternal behavior) and distal (family SES) factors in understanding variation in long-range functional cortical networks in the infant brain. Three key networks—the fronto-parietal network, default mode network, and homologous-interhemispheric connections—were detectable using fNIRS during a passive viewing task at 5 months of age, consistent with hypotheses and previous work (Kelsey et al., 2021a). Contrary to pre-registered hypotheses, however, we observed no direct links between family SES (assessed at the newborn time point as a combination of household income and mothers’ highest level of education) and functional connectivity within these cortical networks at this early age. Moreover, results were not mediated or moderated by sensitive caregiving behavior (a composite of maternal sensitivity and cooperation scales, assessed via observations of mother–infant free-play interactions at 5 months). Crucially, maternal sensitivity showed significant concurrent associations with brain connectivity specifically within the default mode network, providing partial support for hypotheses. We discuss each of these findings in turn, consider their place within the extant literature on functional brain development, and outline promising avenues for future research on the context of early neurodevelopment.

### Characterizing Functional Networks in the Infant Brain

In preliminary analyses, we explored patterns of functional connectivity within and across our pre-defined networks at this time point. At the level of normative development, we found evidence that connectivity within each of our three key infant brain networks (FPN, DMN, and HI) was significantly greater than zero, and that the FPN and DMN showed significantly greater connectivity than a pre-defined non-functional control network (CN) as early as 5 months of age, providing support for our preliminary hypotheses. Connectivity in the HI was similarly higher compared to the CN, but this difference did not survive Bonferroni correction, possibly because both networks involve interhemispheric connections and thus were intercorrelated. This finding was in contrast to previous work using the same cap design and network configuration for the HI and CN in 1-month-old infants (Kelsey et al., 2021a), suggesting that there might be a developmental shift in the association between these two networks from the newborn period to 5 months. Another possible explanation could be that the HI and CN include more long-distance channel pairs than the FPN or DMN, an explanation that was explored further through the creation of a new non-functional random network made up of purely random interhemispheric connections (see Supplementary Material). However, the results of these exploratory analyses suggest that our CN was as non-functional as a purely random network and that differences in connectivity between the functional networks and the CN are unlikely to be driven solely by differences in the distances between the channels in the network. Overall, connectivity in the three networks of interest—FPN, DMN, and HI — did not significantly differ from each other, and this suggests roughly equivalent development of the long-range cortical connections within these functional networks, at least at this early age. Notably, however, longitudinal work shows different developmental trajectories of infant brain networks over the first 12 months of postnatal life, with the DMN showing faster maturation than the FPN (Gao et al., 2015).

Results extend previous work in this sample demonstrating the existence of key functional brain networks as early as the newborn period (Kelsey et al., 2021a) and contribute to a growing body of research demonstrating that these networks are detectable in infancy (Gao et al., 2009, 2017). Yet it is thought that long-range cortical connections in these networks undergo protracted development across childhood and adolescence (Gao et al., 2017), and variability in these developmental pathways are relevant for long-term cognitive development and mental health outcomes (e.g., Ho et al., 2015; Castellanos and Aoki, 2016; Lin et al., 2019). Ongoing work using resting state neuroimaging methods with young infants may thus be considered an important tool for early screening, prevention, and intervention efforts during an important period of development (Gao et al., 2017). Given the high level of experience-dependent plasticity characterizing the first years of life, it is especially important to identify modifiable environmental factors that contribute to individual differences in infants’ neural development; we turn to these findings next.

### Socioeconomic Status: Null Associations With Network Connectivity at 5 Months

At the level of individual differences, SES was not associated with connectivity in any functional brain network at 5 months when compared to the control network, contrary to Hypothesis 1. Findings contrast with previous work in older children showing that SES is related to brain structure and function, particularly within networks involving prefrontal regions implicated in cognitive control (for reviews see Johnson et al., 2016; Hyde et al., 2020; Olson et al., 2021). Note, however, that findings may differ in part because the present study measured somewhat different areas; thus, SES-related differences in connectivity may occur between deeper (subcortical) brain structures that we are unable to assess with fNIRS. In one of the few studies examining SES-related differences in functional connectivity within the first year, Gao et al. (2015) found significant associations between SES (i.e., higher income and maternal education) and greater maturation of the DMN and sensorimotor network among *N* = 65 infants at 6 months of age, but not at any other age points (1, 3, 9, or 12 months). However, we note that these results did not survive multiple comparisons and should be regarded as preliminary.

One explanation is that SES-related effects may emerge only later in development (e.g., at 6 months, or beyond the first year), potentially as the result of cumulative effects of socioeconomic stressors (but see Ramphal et al., 2020 for evidence that SES-related differences in fronto-striatal connectivity are observable in newborns).^1^ For example, one study of 5- to 17-year-old children showed that the magnitude of SES-related effects on children’s brain structure increased with age for temporal and frontal brain regions (Noble et al., 2012), and similar SES by age interactions may characterize the development of brain connectivity. Relatedly, temporal dynamics of SES—such as the timing and chronicity of household financial stress or the instability of resources over the first years of life—may be more important than SES measured at a single time point (see e.g., Lupien et al., 2009; Najman et al., 2010). Long-range cortical networks like the FPN and DMN show particularly protracted development, and further longitudinal work is needed to understand whether, how, and when SES may shape their development.

Beyond developmental effects, it is also possible that the observed null findings stem from inadequate statistical power. While a sensitivity post-hoc power analysis in G*Power indicated that our current sample size would provide enough power to detect a small to medium effect (*f* = 0.28), if effect sizes at this age are quite small, larger sample sizes with a wider range of socioeconomic strata (particularly greater representation of families living in poverty) may be necessary to detect meaningful differences. Further, SES-related effects may be indirect, mediated by factors such as family stress, marital conflict, housing instability, environmental toxins, harsh or neglectful parenting, or lack of access to affordable quality healthcare (Johnson et al., 2016).

### Maternal Sensitivity: Network-Specific Links to the Default Mode Network

Notably, there were no indirect associations of SES with infant functional connectivity via maternal sensitivity, because SES was unrelated to caregiving behavior in the present sample. We interpret this finding as a sign of resilience, suggesting that socioeconomic stress did not compromise these mothers’ ability to provide high-quality care to their infants. Importantly, however, the maternal sensitivity composite was associated with greater connectivity specifically in the default mode network, even when accounting for SES, providing partial support for Hypothesis 2. The DMN is implicated in social cognition (e.g., mentalizing, theory of mind), stimulus-independent thought, and self-referential and introspective processes (for reviews see Spreng and Grady, 2010; Mak et al., 2017; Buckner and DiNicola, 2019; Richardson and Saxe, 2020). A large body of research has shown that sensitive parental care in early development is a robust predictor of related child outcomes, including theory of mind (e.g., Symons and Clark, 2000; Licata et al., 2016), cognitive ability (Landry et al., 2006), and a range of social-emotional competencies (for a review see Deans, 2020). Moreover, attachment theory and research suggest that maternal sensitivity in infancy is a critical building block of children’s mental representations of the self and others (Bowlby, 1969/1982; Ainsworth, 1979; Cassidy and Shaver, 2016; Main et al., 1985). In light of the present findings, we propose that sensitive care may help to organize neural connections that support social cognition, mentalization, and self-representation (“internal working models”; Bowlby, 1969/1982). Thus, functional connectivity of the DMN may be one neural mechanism by which early experiences of sensitive care get “under the skin” to shape later social-cognitive development.

Findings are broadly consistent with research linking caregiving quality to the structure and function of brain regions involved in the DMN. For example, in a sample of 6-month-old infants, maternal sensitivity was associated with greater connectivity of the hippocampus with the medial prefrontal cortex and superior and middle temporal cortex; sensitivity also predicted the development of limbic brain structures involved in social cognition, emotion regulation, and autobiographical memory (Rifkin-Graboi et al., 2015) (note that in the present study, we were unable to assess connectivity with inner brain structures due to the limitations of fNIRS). Further, 5-year-old children who experienced greater frequency of maternal touch showed greater resting state activity and connectivity in regions of the DMN including the superior temporal sulcus and temporo-parietal junction (Brauer et al., 2016). In contrast, early life exposure to maternal depression and insensitive caregiving has been linked to disrupted brain development, specifically reduced DMN connectivity in preadolescence (Zeev-Wolf et al., 2019, 2022). Relatedly, resting state connectivity in the DMN is lower among adults who report experiencing early-life trauma (i.e., childhood abuse; Bluhm et al., 2009). The present findings suggest that normative variation in maternal sensitivity is related to the functioning of the DMN as early as 5 months of age, adding to a thriving literature on parent-child interactions as a vital context for early neurodevelopment (see Ilyka et al., 2021).

Importantly, maternal sensitivity did not interact with SES to predict infant functional connectivity, and thus, Hypothesis 3 was not supported. As with the null main effects of SES on functional connectivity, it is possible that interactions with maternal sensitivity may emerge later in development, given experimental findings demonstrating that interventions to enhance caregiving quality can successfully mitigate the impact of early deprivation on brain structure and function in middle childhood (Sheridan et al., 2012), and protect against the detrimental effects of childhood poverty on brain development in adolescence (Brody et al., 2017).

It is interesting to note that at the bivariate level, DMN connectivity was more strongly related to the *maternal cooperation* dimension of sensitivity—that is, mothers’ support for infants’ autonomous activity and exploration, with minimal parental interference. Previous work has linked maternal intrusiveness (i.e., low cooperation) to infants’ ERP responses to emotional vocalizations (Huffmeijer et al., 2020); further, observed maternal autonomy support in infancy has been shown to be an especially strong predictor of children’s later self-regulation (Bernier et al., 2010). In the present study, it is possible that the observed association between the sensitivity composite and DMN connectivity is driven by maternal cooperation because cooperation allows the infant greater freedom to explore the social world uninterrupted, fostering greater autonomy and faster maturation of neural networks implicated in social cognition.

### Strengths, Limitations, and Future Directions

The present study is first to our knowledge to examine the unique and interactive effects of socioeconomic status and observed sensitive caregiving on infant resting state functional connectivity as early as 5 months of age. Strengths of the study include the use of a classic gold-standard observational measure of sensitive caregiving (Ainsworth, 1969) in combination with cutting-edge fNIRS techniques with infants in the first months of life. Moreover, hypotheses were pre-registered and tested using stimuli specifically designed for infants with non-invasive neuroimaging methods. Finally, this work included examination of multiple infant brain networks, with a conservative point of comparison to the control network, which has rarely been included in previous work.

Yet findings should also be considered in light of the study’s limitations, which point to several promising avenues for future research. First, the study’s correlational design precludes causal inference; thus, it is possible that variation in brain connectivity underlies infant behaviors that elicit specific types of maternal caregiving (note that in the present sample, however, maternal sensitivity was unrelated to infants’ negative affect, ruling out a potential confound). The field is ripe for experimental work that examines (a) how infant brain development may be positively impacted by *social policies and programs to reduce poverty* (e.g., Noble and Giebler, 2020) and (b) *interventions to promote sensitive caregiving* (e.g., Valadez et al., 2020), as well as the role of developmental timing in enhancing the efficacy of such interventions (e.g., Vanderwert et al., 2016).

Second, the final analytic sample was small, United States-based, mother-centric, and predominantly White, limiting statistical power to detect smaller effect sizes as well as generalizability to other cultures, family structures, and childrearing contexts. Relatedly, although families’ SES showed substantial variability and normal distribution in our sample, it is possible that effects on infant functional connectivity may only be detected at the extremes (i.e., in samples with a larger proportion of families experiencing poverty). Future research in larger samples should examine (c) whether links of SES and sensitive caregiving to infant brain connectivity are *moderated* by factors such as infants’ biological sensitivity to context (e.g., temperamental reactivity, genetic markers; Boyce and Ellis, 2005; Belsky and Pluess, 2009); (d) which specific *dimensions of SES* may be most relevant to infant brain development (e.g., family stress, food insecurity, variation in cognitive stimulation, neighborhood-level poverty and violence, environmental hazards, exposure to racism, subjective social status; for discussion see Antonoplis, in press); (e) the unique and interactive contributions of *paternal and alloparental care* (Feldman et al., 2019; for evidence linking paternal sensitivity to infant brain structure, see Sethna et al., 2019); and (f) whether results converge or differ *cross-culturally*, particularly which features of caregiving behavior are most promotive of healthy brain development for children within specific socioecological contexts [for discussion see Hyde et al. (2020)].

Third, although fNIRS provides a non-invasive, infant-friendly technique to assess neural activity early in ontogeny, the methodology captures activity only at the surface of the cortex; thus, future work is needed to examine corticolimbic pathways, particularly connections with environmentally sensitive structures such as the amygdala (e.g., Gee et al., 2013; Cohodes et al., 2021). Additionally, our pre-registered processing pipeline included motion correction, following recent recommendations from multiple infant fNIRS researcher labs (e.g., Blanco et al., 2021; Baek et al., 2022); as a results, however, findings may differ somewhat from those of research labs using motion rejection (e.g., Molavi and Dumont, 2012; Behrendt et al., 2018; Bulgarelli et al., 2019, 2020). Both approaches have strengths and weaknesses, and future work may shed light on best practices for detecting long-range functional networks in this age group.

Finally, we focused analyses of infant neural connectivity on the 5-month time point (an *a priori*, pre-registered decision), but future longitudinal analyses may shed light on whether findings at this time point are generalizable to other age groups, as well as how SES and maternal sensitivity shape developmental trajectories of infant brain development over time. Indeed, our own planned future work envisions following this sample into the toddler and preschool years. This is important because studying the first 5 months of life may not provide enough time for SES and maternal sensitivity to be biologically embedded in terms of postnatal development (Hanson et al., 2013; Gao et al., 2015; Brito et al., 2016). Longitudinal investigations may be especially powerful in testing (g) the role of *developmental timing* as well as (h) *stability* and *chronicity* of SES-related stressors and features of the caregiving environment in shaping infant brain connectivity. Such work should further investigate (i) the *mechanisms* by which SES and sensitive caregiving may (perhaps indirectly) relate to infant brain development, such as the calibration of the HPA-axis (Holochwost et al., 2020), epigenetic modulation of neurodevelopment (Conradt et al., 2016; Krol et al., 2019a), and variation in the gut-brain axis (Kelsey et al., 2019a,2021b; Flannery et al., 2020).

### Conclusion

The present study advances current understanding of the developmental origins of brain connectivity, as well as sources of individual variation in the first months of life. Contrary to expectations, there were no significant associations between family socioeconomic status and infant brain connectivity at 5 months of age in the present community sample; however, we underscore that such associations may emerge later in development. We did find evidence for network-specific associations of maternal sensitivity with connectivity in the default mode network, a neural network underlying fundamental social-emotional competencies such as theory of mind, mentalizing, and representations of the self and others (Buckner and DiNicola, 2019). Findings point to a potential early-emerging neural mechanism linking early caregiving experiences to later social-emotional functioning (e.g., Licata et al., 2016).

Critically, looking beyond the present work, there is ample evidence that socioeconomic stress and inequality is detrimental to family functioning and other aspects of brain development across childhood (Shonkoff et al., 2000; Blair and Raver, 2016). Further, even if future research continues to find no effects of SES on infant brain connectivity specifically, policies to reduce family poverty and address economic inequities remain not only ethical and just, but also scientifically sound for promoting multiple additional domains of healthy child development (Aber et al., 2012; Perrin et al., 2020). The present work adds to a growing body of evidence that high-quality relationships with responsive caregivers are a key predictor of brain development as early as infancy, even when accounting for SES. Thus, programs and policies that reduce family stress, strengthen supports for caregivers to foster sensitive caregiving, and work to create health-promotive socioecological contexts for early development are important for nurturing the developing brain (Brody et al., 2017).

## Data Availability Statement

The raw data supporting the conclusions of this article will be made available by the authors, without undue reservation.

## Ethics Statement

The studies involving human participants were reviewed and approved by University of Virginia’s Institutional Review Board for Health Sciences Research. Written informed consent was obtained from the individual(s), and minor(s)’ legal guardian/next of kin, for the publication of any potentially identifiable images or data included in this article.

## Author Contributions

CK and TG contributed to the conception and design of the original longitudinal study. CK collected the data and processed the fNIRS data. JS led behavioral coding of maternal sensitivity and wrote the first draft of the discussion. JC performed the statistical analysis, wrote the first draft of the introduction, and wrote the first draft of the results. JS and CK wrote the first draft of the “Materials and Methods”. All authors contributed to conception of the specific research questions addressed in the current manuscript, manuscript revision, read, and approved the submitted version.

## Conflict of Interest

The authors declare that this study received funding from Danone North America. The funder was not involved in the study design, collection, analysis, interpretation of data, the writing of this article or the decision to submit it for publication.

## Publisher’s Note

All claims expressed in this article are solely those of the authors and do not necessarily represent those of their affiliated organizations, or those of the publisher, the editors and the reviewers. Any product that may be evaluated in this article, or claim that may be made by its manufacturer, is not guaranteed or endorsed by the publisher.

## Footnotes

1 Note that the sample reported by Ramphal et al. (2020) included preterm infants.
