# Supplementary material for: Examining the Role of Socioeconomic Status and Maternal Sensitivity in Predicting Functional Brain Network Connectivity in 5-Month-Old Infants
Source: Front Neurosci. 2022 Jun 10;16:892482. doi: 10.3389/fnins.2022.892482 (PMC9226752; doi:10.3389/fnins.2022.892482)
Supplement: Supplementary file 1 [file Data_Sheet_1.pdf]

Supplementary Materials

Adjusted Models Controlling for Number of Children in Household

As part of the parent questionnaire at the 1-month time point, mothers were asked to report the number of siblings their infant had ("How many other biological siblings does the child have?"). The first answer option ("0, only child") was subsequently coded as a 1, "1 sibling" was coded as a 2, and so on. While the original question asked about number of siblings, the data reflect the number of children in the household. Since this variable was significantly correlated with maternal education, we included it as a covariate in all our analyses involving our SES composite variable. The results of these adjusted models are reported below.

SES and Functional Connectivity

For the FPN, when number of children was included as a covariate, there was a significant within-subject effect of network [$F_{(1, 45)} = 4.39$, $p = .042$, $\eta^2_{partial} = 0.09$], but the network by SES interaction was not significant ($p = .281$), nor was the network by number of children interaction ($p = .774$). There were no significant between-subjects effects of SES ($p = .455$) or number of children ($p = .709$).

For the DMN, when number of children was included as a covariate, there was a significant within-subject effect of network [$F_{(1, 46)} = 4.31$, $p = .044$, $\eta^2_{partial} = 0.09$], but the network by SES interaction was not significant ($p = .236$), nor was the network by number of children interaction ($p = .925$). There were no significant between-subjects effects of SES ($p = .351$) or number of children ($p = .693$).

For the HI, when number of children was included as a covariate, there was no significant within-subject effect of network ($p = .845$), network by SES interaction ($p = .277$), or network by

number of children interaction (p = .284). There were no significant between-subjects effects of SES (p = .233) or number of children (p = .750).

Maternal Sensitivity as a Mediator of the Association Between SES and Functional Connectivity

When number of children was included as a covariate in the bootstrapped mediation models, the link between SES and maternal sensitivity composite remained non-significant (average b = 0.16, all ps > .330), and thus there were no indirect associations of SES with infant functional connectivity. However, in all models, number of children was significantly negatively associated with maternal sensitivity (average b = -0.73, p = .010). Maternal sensitivity composite remained significantly positively associated with DMN connectivity (b = 0.04, p = .027), even when controlling for SES and number of children.

Maternal Sensitivity as a Moderator of the Association Between SES and Functional Connectivity

When number of children was included as a covariate in the bootstrapped moderation models, SES did not interact with maternal sensitivity composite to predict functional connectivity in any network, and thus no models were significant (all ps > .482).

Exploratory Analyses

Correlations with Number of Usable Channels

A series of Spearman's rho correlations was used to identify significant associations between the main variables of interests (household income, maternal education, maternal sensitivity, maternal cooperation, FPN connectivity, DMN connectivity, HI connectivity, CN connectivity) and the number of usable fNIRS channels. Number of usable channels was positively correlated with SES [Income ($r_{(48)}$ = .42, p = .003); Education ($r_{(48)}$ = .29, p = .040)]

and the CN oxyHb ($r_{(48)}$ = .35, p = .013), but not maternal sensitivity or any of the functional oxyHb networks (all ps > .093). Number of usable channels was also positively correlated with most of the deoxyHb networks [FPN ($r_{(48)}$ = .37, p = .010); DMN ($r_{(48)}$ = .41, p = .004); CN ($r_{(48)}$ = .33, p = .020)] but not the HI deoxyHb network ($r_{(48)}$ = .21, p = .146).

Mediation Model with Control Network

In the unadjusted model, SES did not predict maternal sensitivity composite (b = 0.0003, p = .999), so the indirect association with the CN was not significant. SES did have a marginally significant link to CN connectivity (b = 0.02, p = .082), but maternal sensitivity did not (b = -0.003, p = .757), demonstrating specificity of the link between maternal sensitivity and the DMN. In the adjusted model, number of children significantly predicted maternal sensitivity (b = -0.72, p = .010), but SES did not (b = -0.17, p = .338). There were no significant associations with CN connectivity (all ps > .143).

Moderation Model with Control Network

In both the unadjusted and adjusted models, SES did not interact with maternal sensitivity composite to predict functional connectivity in the CN (all ps > .796).

DeoxyHb Results

Preliminary Analyses

A series of Spearman's rho correlations was used to identify significant associations between the main variables of interests (household income, maternal education, maternal sensitivity scale, maternal cooperation scale, FPN deoxyHb connectivity, DMN deoxyHb connectivity, HI deoxyHb connectivity, CN deoxyHb connectivity) and potential covariates (FC seconds, infant sex assigned at birth, mother race, infant race, number of children) (see Supplementary Table 2). Only covariates that were significantly associated with a main variable

of interest were included as covariates in subsequent analyses. Significant correlations that have already been reported and accounted for in the main text will not be mentioned below.

HI deoxyHb connectivity was significantly associated with FPN deoxyHb connectivity ($r_{(48)} = .528$, $p < .001$), DMN deoxyHb connectivity ($r_{(48)} = .282$, $p = .047$), and CN deoxyHb connectivity ($r_{(48)} = .419$, $p = .002$). DMN deoxyHb connectivity was also significantly associated with CN deoxyHb connectivity ($r_{(48)} = .408$, $p = .003$). Regarding potential covariates, FPN deoxyHb connectivity was significantly associated with maternal education ($r_{(48)} = .340$, $p = .016$) and infant race such that White infants exhibited greater connectivity ($r_{(48)} = -.292$, $p = .039$), DMN deoxyHb connectivity was significantly associated with infant sex such that female infants exhibited greater connectivity ($r_{(48)} = .278$, $p = .050$), and HI deoxyHb connectivity was significantly associated with mother race such that infants whose mothers are White exhibited greater connectivity ($r_{(48)} = -.326$, $p = .021$). Notably, maternal sensitivity scale, maternal cooperation scale, and FC seconds were not significantly associated with any of the deoxyHb functional connectivity measures (all *p*s > .195).

Supplementary Table 3 shows the correlation values between all oxyHb and deoxyHb functional connectivity networks.

DeoxyHb Functional Connectivity Across Networks

First, a series of one-sample *t*-tests was conducted to assess whether Fisher-transformed correlations between individual channels within each pre-defined network differed from zero. Results for deoxyHb channels are shown in Figure 2 and Supplementary Table 1. Next, a series of one-sample *t*-tests was conducted to assess network-level connectivity by combining across all channels of interest. All four deoxyHb networks were greater than zero [FPN: $t_{(49)} = 7.18$, $p <$

.001, $d = 0.18$; DMN: $t_{(49)} = 7.34$, $p < .001$, $d = 0.14$; HI: $t_{(49)} = 6.53$, $p < .001$, $d = 0.14$; CN: $t_{(49)} = 5.24$, $p < .001$, $d = 0.12$; see Supplementary Figure 2].

To examine differences in overall connectivity levels across the four networks, an omnibus repeated measures ANOVA was conducted with network type (FPN, DMN, HI, CN) as a within-subject factor. The analysis revealed a significant within-subject effect across network types [$F_{(3,\ 147)} = 5.75$, $p < .001$, $\eta^2_{\text{partial}} = 0.11$]. Post hoc analysis with a Bonferroni adjustment revealed that FPN deoxyHb connectivity ($M = 0.18$, $SD = 0.18$) was significantly greater than HI deoxyHb connectivity ($M = 0.13$, $SD = 0.14$) {pairwise comparison ($M_{FPN} – M_{HI} = 0.06$ [95% CI, 0.01–0.11], $p = .021$)} and CN deoxyHb connectivity ($M = 0.09$, $SD = 0.12$) {pairwise comparison ($M_{FPN} – M_{CN} = 0.09$ [95% CI, 0.02–0.16], $p = .005$)}. DMN deoxyHb connectivity ($M = 0.14$, $SD = 0.14$) was also significantly greater than CN deoxyHb connectivity {pairwise comparison ($M_{DMN} – M_{CN} = 0.05$ [95% CI, 0.0004–0.10], $p = .047$)}. However, there was no significant difference between HI deoxyHb connectivity and CN deoxyHb connectivity {pairwise comparison ($M_{HI} – M_{CN} = 0.03$ [95% CI, -0.02–0.08], $p = .403$)}. There were no other significant differences in deoxyHb network connectivity (all *p*s > .999; see Supplementary Figure 2).

SES and DeoxyHb Functional Connectivity

To assess how differences in family socioeconomic status were associated with infant functional connectivity, separate repeated measures ANOVAs were conducted for each functional network of interest (FPN, DMN, HI). For each rmANOVA, the deoxyHb functional network of interest and the CN were entered as within-subject factors, and a dichotomized SES score was entered as a between-subjects factor.

In the unadjusted models, there was a significant within-subject effect of network for the FPN [$F_{(1, 48)} = 12.47$, $p < .001$, $\eta^2_{partial} = 0.21$] and the DMN [$F_{(1, 48)} = 7.54$, $p = .008$, $\eta^2_{partial} = 0.14$], but not for the HI [$F_{(1, 48)} = 3.44$, $p = .070$, $\eta^2_{partial} = 0.07$]. In the adjusted models, there was a significant within-subject effect of network for all three functional networks {FPN: [$F_{(1, 45)} = 8.45$, $p = .006$, $\eta^2_{partial} = 0.16$]; DMN: [$F_{(1, 45)} = 4.30$, $p = .044$, $\eta^2_{partial} = 0.09$]; HI: [$F_{(1, 45)} = 6.80$, $p = .012$, $\eta^2_{partial} = 0.13$]}. However, in all adjusted and unadjusted models, SES was not significantly associated with connectivity in any functional brain network, regardless of covariates or analytic approach (all *p*s > .134).

Maternal Sensitivity as a Mediator of the Association Between SES and DeoxyHb Functional Connectivity

In the unadjusted models, SES was not associated with maternal sensitivity composite, and thus no mediation models were significant (all *p*s > .999). However, SES was directly associated with FPN deoxyHb connectivity specifically ($b = 0.03$, $p = .045$, see Supplementary Figure 3).

In the adjusted models, the covariates of the SES component variables, namely household income and maternal education, and each deoxyHb functional network were included in the respective models. In all models, number of children was significantly associated with maternal sensitivity (average $b = -0.72$, all *p*s < .020), but SES was not (all *p*s > .370). For the FPN, only infant race was directly associated with FPN deoxyHb connectivity ($b = -0.12$, $p = .042$). For the DMN, infant sex was associated with maternal sensitivity ($b = 1.26$, $p = .025$), but there were no significant associations with DMN deoxyHb connectivity (all *p*s > .136). For the HI, only mother race was significantly associated with HI deoxyHb connectivity such that infants whose mothers are White exhibited greater connectivity ($b = -0.13$, $p = .028$).

Finally, we looked at a model where the CN was the deoxyHb network of interest. In both the unadjusted and adjusted models, SES was not associated with maternal sensitivity (b = 0.0003, p = .999), so the mediation models were not significant, and there were no significant associations of SES or maternal sensitivity with CN deoxyHb connectivity (all ps > .340).

Maternal Sensitivity as a Moderator of the Association Between SES and DeoxyHb Functional Connectivity

In both the unadjusted and adjusted moderation models, SES did not interact with maternal sensitivity composite to predict deoxyHb functional connectivity in any network, and thus no moderation models were significant (all ps > .104). Additionally, we tested a model where the CN was the deoxyHb network of interest, and SES also did not interact with maternal sensitivity to predict functional connectivity in this network (p = .140).

Income-to-Number-of-Children Ratio as the SES Variable

As an alternative to including number of children as a covariate in our analyses involving SES, we created a new SES variable equal to annual household income divided by the number of children in the household to be used in place of our SES composite variable in all main and exploratory analyses. Using this new SES variable in place of our SES composite variable did not alter the significance levels of any of our main results. However, it did impact a few of our exploratory analyses involving deoxyHb functional connectivity. When assessing the association between SES and deoxyHb functional connectivity, there was no longer a significant within-subject effect of network for the DMN in the adjusted model [$F_{(1, 47)} = 3.58$, $p = .065$, $\eta^2_{\text{partial}} = 0.07$]. When testing our mediation model for the FPN, SES was no longer significantly associated with FPN deoxyHb connectivity in the unadjusted model (b = 0.01, p = .179).

Test of Laterality Effects for the FPN and DMN

To examine if there were laterality effects for the FPN and DMN, we split these networks into left and right hemispheres and compared the levels of connectivity between them using a series of repeated measures ANOVAs. For the DMN, there was a significant laterality effect for oxyHb connectivity, such that the right DMN exhibited significantly greater connectivity on average (*M* = 0.25, *SD* = 0.25) compared to the left DMN (*M* = 0.17, *SD* = 0.22) [$F_{(1, 49)} = 4.64$, $p = .036$, $\eta^2_{partial} = 0.09$]. This same laterality effect was also present for DMN deoxyHb connectivity [Right DMN: *M* = 0.21, *SD* = 0.18; Left DMN: *M* = 0.07, *SD* = 0.16; $F_{(1, 49)} = 26.28$, $p < .001$, $\eta^2_{partial} = 0.35$]. For the FPN, there was not a significant laterality effect for oxyHb connectivity [Right FPN: *M* = 0.25, *SD* = 0.27; Left FPN: *M* = 0.19, *SD* = 0.23; $F_{(1, 48)} = 1.84$, $p = .182$, $\eta^2_{partial} = 0.04$]. However, there was a laterality effect for deoxyHb connectivity, such that the right FPN exhibited significantly greater connectivity (*M* = 0.23, *SD* = 0.23) compared to the left FPN (*M* = 0.13, *SD* = 0.20) [$F_{(1, 49)} = 9.24$, $p = .004$, $\eta^2_{partial} = 0.16$]. These findings suggest that in general, infants in our sample had increased levels of functional connectivity in the right hemisphere compared to the left hemisphere of the DMN, and to a lesser extent the FPN.

Follow-up Analyses Using a Random Network Approach

One possible explanation for the differences in connectivity between the FPN/DMN and the CN, but not between the HI and the CN, is that both the HI and CN include more long-distance channel pairs than the FPN or DMN, although the lengths of the connections within the HI do vary greatly with some channel pairs located relatively close together. To test this explanation, we created a new non-functional random network (RN, see Supplementary Figure 4A) made up of purely random connections (and notably, several with shorter distances) to use in place of our control network to see if that changed the outcomes of any of our analyses. First, we

conducted a series of one-sample t-tests to assess whether Fisher-transformed correlations between individual channels within the RN differed from zero. As shown in Supplementary Figure 4B, these analyses identified only three channel pairs in the RN that had functional connectivity levels that were significantly different from zero for either oxyHb, deoxyHb, or both (see Supplementary Table 1 for a full summary of the results). Notably, this was a much smaller percentage of connections that differed from zero (RN: 19% of channels) compared to all other pre-defined networks (FPN: 50% of channels; DMN: 50% of channels; HI: 43% of channels; CN: 50% of channels).

However, when the RN was examined at the network level rather than at the channel level, the results were very similar to our original CN. For instance, like the CN, the overall level of connectivity in the RN was significantly greater than zero [$t_{(49)} = 4.95$, $p < .001$, $d = 0.14$], and there was no difference in connectivity between the original CN ($M = 0.10$, $SD = 0.15$) and the new RN ($M = 0.10$, $SD = 0.14$) [$t_{(49)} = -0.33$, $p = .741$, $d = 0.16$]. Additionally, we conducted a rmANOVA with network type (FPN, DMN, HI, CN, RN) as a within-subject factor to examine differences in overall connectivity across the networks. Our findings were very similar to our originally reported results, in that there was still a significant within-subject effect of network [$F_{(4, 192)} = 8.23$, $p < .001$, $\eta^2_{\text{partial}} = 0.15$]. Specifically, FPN connectivity was significantly greater than CN connectivity ($M_{FPN} - M_{CN} = 0.12$ [95% CI, 0.03–0.22], $p = .004$) and RN connectivity ($M_{FPN} - M_{RN} = 0.12$ [95% CI, 0.03–0.22], $p = .004$), DMN connectivity was significantly greater than CN connectivity ($M_{DMN} - M_{CN} = 0.11$ [95% CI, 0.02–0.20], $p = .005$) and RN connectivity ($M_{DMN} - M_{RN} = 0.11$ [95% CI, 0.03–0.19], $p = .002$), but HI connectivity did not differ from CN connectivity ($M_{HI} - M_{CN} = 0.06$ [95% CI, -0.01–0.13], $p = .132$) or RN connectivity ($M_{HI} - M_{RN} = 0.06$ [95% CI, -0.01–0.13], $p = .099$). Taken together, these results provide strong evidence

that at the network level, our CN was as non-functional as a network made up of completely random channel pairs with a variety of short-range and long-range connections. Therefore, the original CN is serving its intended purpose as a non-functional control network or baseline, and differences in connectivity between the functional networks and the CN are unlikely to be driven solely by differences in the distances between the channels.

Supplementary Figures and Tables

Supplementary Figure 1

Histogram of Amount of Functional Connectivity Data for Included and Excluded Participants

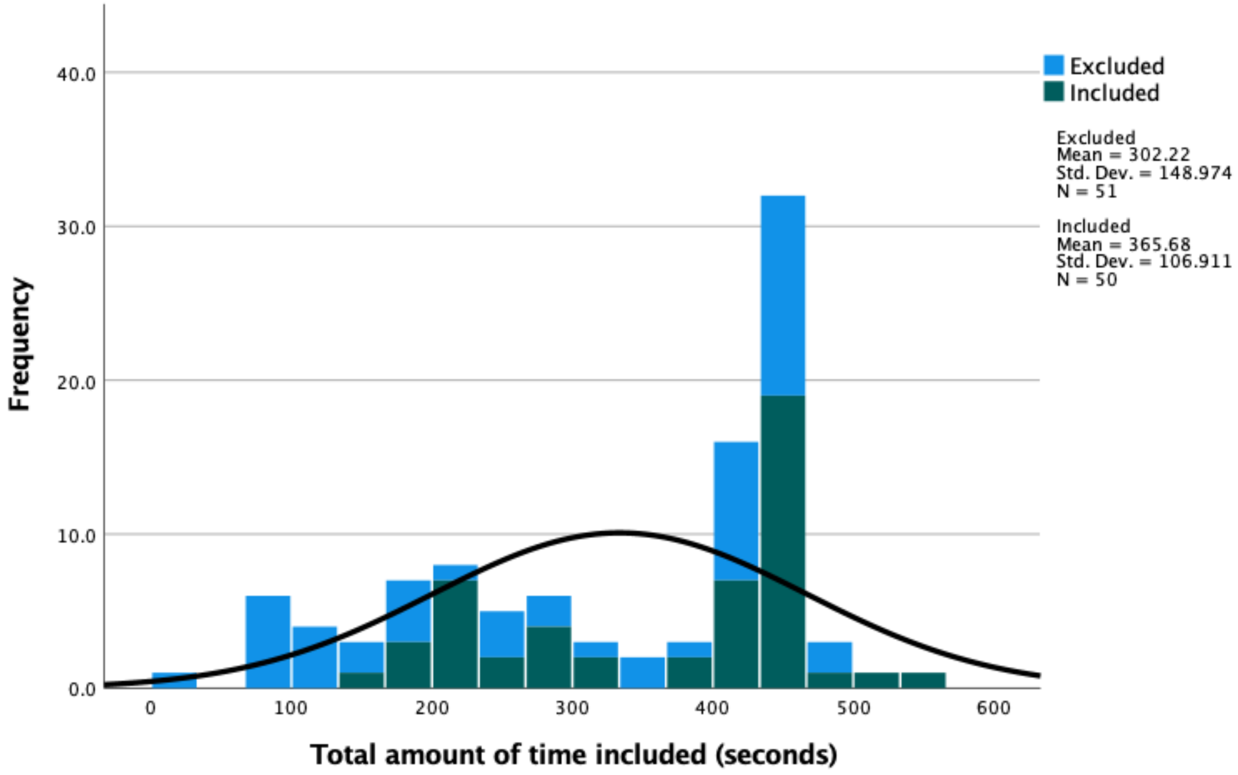

Note. Bars indicate the total amount of functional connectivity data for included and excluded participants. The means and standard deviations for each group are also reported. Curved line represents a normal distribution.

Supplementary Figure 2

Infant Functional Connectivity by Network at 5 Months – DeoxyHb Connectivity

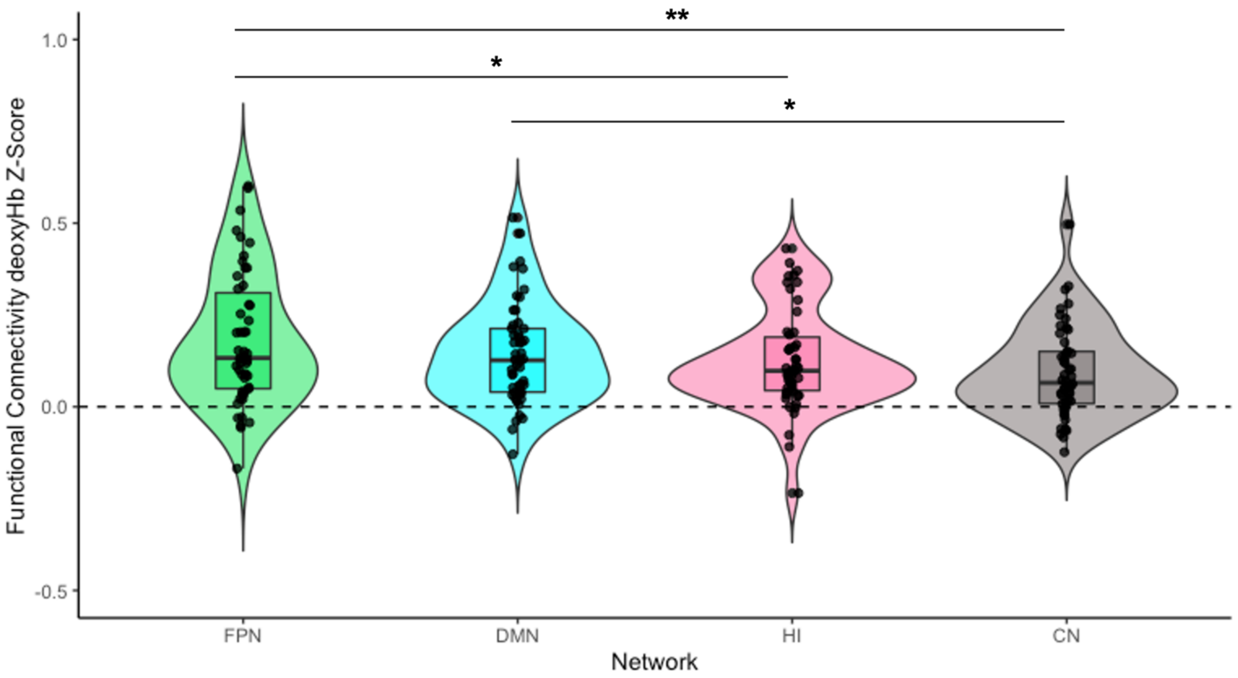

Note. This figure shows the average levels of functional connectivity (deoxyHb) for each network. The boxplot horizontal lines from bottom to top reflect values for the lower quartile, median, and upper quartile respectively. FPN, fronto-parietal network; DMN, default mode network; HI, homologous-interhemispheric connections; CN, control network. $N = 50$.

*$p < .05$, **$p < .01$.

Supplementary Figure 3

Association Between SES Composite and Infant FPN DeoxyHb Connectivity at 5 Months

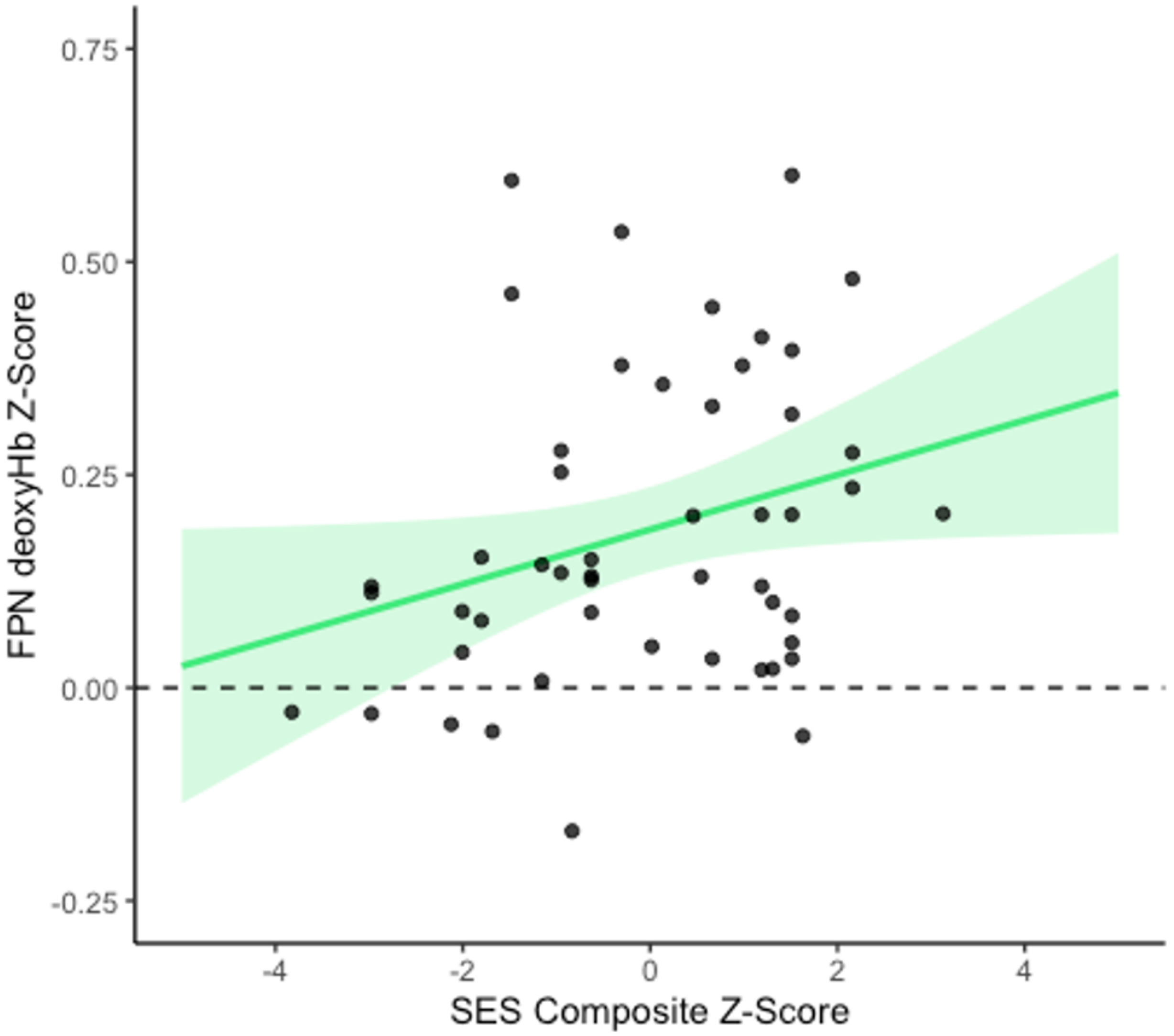

Note. This figure shows the unadjusted relation between SES composite Z-score and FPN functional connectivity (deoxyHb) Z-scores. The green shaded area represents the upper and lower bounds of the mean 95% confidence interval for the raw data. $N = 50$.

Supplementary Figure 4

Schematic of Random Network Configuration and Channel Pair Connectivity

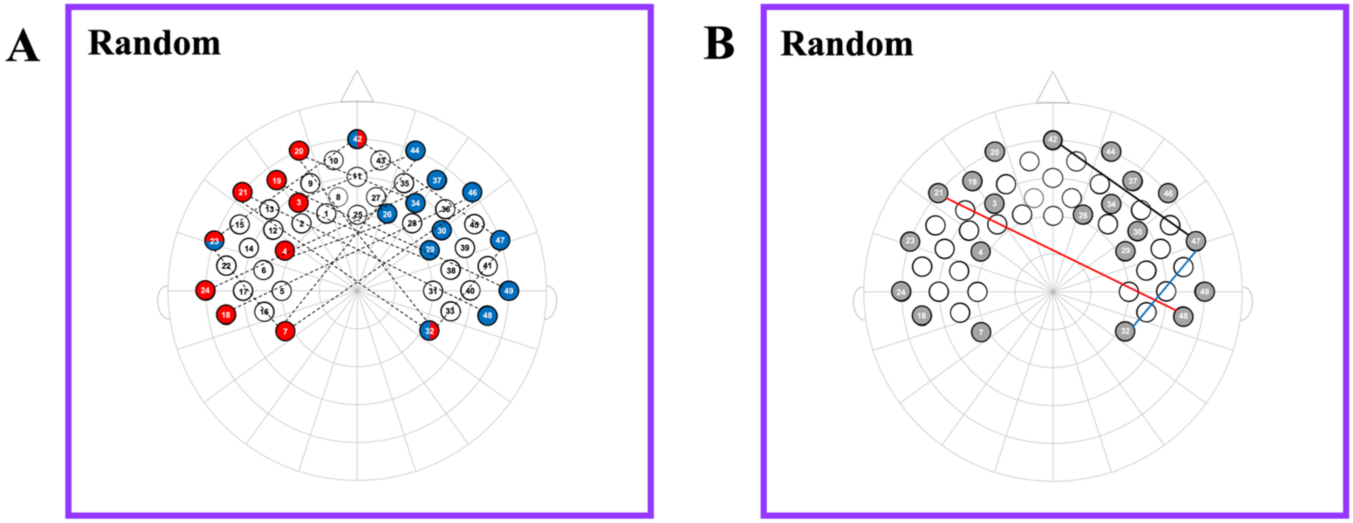

Note. Panel A shows the configuration for the new random network (RN) in a 2-dimentional 10-20 system layout. The network consists of the average of the correlations between each red-blue channel pair that is connected by a dotted line. Panel B shows the channel pairs in the random network with functional connections that are significantly different from zero. Connections in red, blue, and black represent significant changes between the channels for oxyHb, deoxyHb, and both oxy and deoxyHb, respectively.

Supplementary Table 1

Descriptive Statistics for fNIRS Channels

	OxyHb				DeoxyHb			
Channel Pairs	*n*	*M*	*SD*	2-sided *p*	*n*	*M*	*SD*	2-sided *p*
FPN								
2--7	61	0.27	1.48	.164	78	0.17	1.22	.216
2--16	62	0.11	0.37	.027*	78	0.07	0.34	.060
3--7	66	0.25	1.48	.177	78	0.22	1.23	.123
3--16	66	0.02	0.36	.629	78	0.06	0.32	.108
7--13	64	0.21	2.02	.414	78	0.36	1.77	.076
13--16	**64**	**0.18**	**0.34**	**< .001***	78	0.11	0.31	.002*
28--32[†]	35	<0.01	0.50	.968	78	0.90	0.28	.005*
28--33	66	0.28	1.94	.254	78	0.45	1.72	.023*
32--34[†]	34	-0.09	0.40	.201	78	0.05	0.25	.073
32--36	35	-0.07	0.42	.320	78	<-0.01	0.28	.996
33--34	69	0.66	2.21	.016*	**78**	**0.87**	**2.11**	**< .001***
33--36	71	0.62	2.23	.021*	**78**	**0.87**	**2.24**	**< .001***
DMN								
10--18	47	0.39	1.95	.176	78	0.19	1.50	.275
10--22	61	-0.02	1.41	.902	78	0.04	1.20	.754
10--23	55	-0.18	1.48	.380	78	-0.07	1.19	.615
10--24	49	0.06	0.49	.421	78	-0.01	0.45	.865
10--41	53	0.10	0.46	.118	78	0.04	0.33	.325
10--47	54	0.19	0.51	.007*	78	0.05	0.51	.430
10--48	55	0.25	1.04	.084	78	0.20	0.91	.058
10--49	56	0.13	0.41	.027*	**78**	**0.17**	**0.35**	**< .001***
18--42	39	-0.09	2.01	.788	78	-0.04	1.35	.811
18--43	38	0.07	0.44	.304	78	0.07	0.36	.083
22--42	55	0.25	1.32	.164	78	0.17	0.97	.130
22--43	54	0.15	0.41	.008*	78	0.09	0.41	.067
23--42	51	0.10	0.75	.356	78	0.07	0.68	.394
23--43	52	0.28	1.32	.127	78	-0.08	0.99	.492
24--42	43	0.31	1.85	.278	78	0.17	1.25	.226
24--43	42	0.11	0.33	.030*	78	-0.04	0.33	.337
41--42	**56**	**0.51**	**0.74**	**< .001***	**78**	**0.34**	**0.63**	**< .001***
41--43	55	0.25	0.57	.002*	**78**	**0.20**	**0.49**	**< .001***
42--47	**54**	**0.36**	**0.64**	**< .001***	**78**	**0.26**	**0.44**	**< .001***
42--48	53	0.17	0.43	.005*	78	0.08	0.40	.090
42--49	**54**	**0.23**	**0.43**	**< .001***	78	0.12	0.35	.002*
43--47	**54**	**0.28**	**0.50**	**< .001***	78	0.11	0.39	.020*
43--48	52	0.10	0.41	.074	78	0.13	0.42	.010*
43--49	54	0.54	1.33	.004*	78	0.18	1.05	.143
HI								
1--26	66	0.34	1.41	.056	78	0.25	1.14	.052
2--28	59	0.46	1.75	.046*	78	0.25	1.22	.069

	OxyHb				DeoxyHb			
Channel Pairs	*n*	*M*	*SD*	2-sided *p*	*n*	*M*	*SD*	2-sided *p*
3--34	66	0.19	1.93	.429	78	0.24	1.59	.189
4--29	8	0.23	0.30	.062	78	0.01	0.24	.649
5--31	8	0.22	0.22	.026*	78	0.08	0.30	.024*
6--38†	45	0.26	1.83	.339	78	0.26	1.38	.099
7--32	35	0.05	0.31	.309	78	0.03	0.25	.294
9--35	67	-0.08	1.40	.655	78	0.28	1.23	.049*
12--30	8	0.24	0.32	.076	78	0.04	0.26	.151
13--36	67	0.44	1.62	.280*	78	0.32	1.46	.053
14--39	**45**	**0.19**	**0.37**	**.001***	78	0.08	0.37	.059
15--45†	49	-0.04	0.33	.434	78	0.07	0.31	.063
16--33	68	-0.08	1.38	.653	78	-0.06	1.42	.730
17--40	44	0.09	0.39	.120	78	0.09	0.29	.009*
18--48	42	-0.13	1.15	.467	78	0.02	0.97	.890
19--37	37	0.03	0.38	.661	78	0.07	0.30	.048*
20--44	39	0.01	2.06	.981	78	0.17	1.42	.287
21--46	46	0.05	0.23	.181	78	0.11	0.30	.002*
22--41	52	0.10	0.30	.019*	78	0.06	0.31	.072
23--47	50	0.07	0.43	.268	78	0.01	0.34	.709
24--49	44	0.09	0.34	.072	78	<0.01	0.26	.936
CN								
15--41	55	0.11	0.33	.017*	78	0.11	0.30	.002*
15--48†	59	0.04	0.33	.316	78	0.08	0.31	.026*
15--49	58	0.07	0.29	.074	78	0.05	0.31	.119
18--37	37	-0.09	0.97	.560	78	<-0.01	0.69	.971
18--45†	41	0.52	1.74	.065	78	0.44	1.44	.008*
18--46	40	0.05	1.36	.812	78	0.08	1.05	.503
19--41	36	-0.02	0.43	.766	78	0.11	0.40	.016*
19--48	42	-0.12	1.05	.446	78	-0.04	0.72	.624
19--49	43	0.41	1.86	.151	78	0.19	1.25	.176
21--41	53	0.05	0.33	.244	78	0.03	0.36	.530
21--48	52	0.13	0.36	.012*	78	0.08	0.36	.064
21--49	52	0.06	0.39	.249	78	0.09	0.27	.005*
22--37	47	0.07	0.28	.101	78	0.08	0.31	.023*
22--45	46	0.39	1.89	.163	78	0.35	1.30	.019*
22--46	47	0.06	0.23	.105	78	0.12	0.34	.002*
24--37	36	0.02	0.34	.703	78	<0.01	0.23	.944
24--45	37	-0.22	1.73	.438	78	-0.16	1.19	.240
24--46	36	0.04	0.30	.410	78	-0.04	0.26	.200
RN								
3--29	8	0.22	0.32	.092	78	-0.01	0.23	.769
3--44	51	0.29	1.61	.199	78	-0.04	1.23	.757
4--26	54	0.08	0.46	.207	78	0.08	0.45	.123
7--23	58	0.19	1.80	.423	78	-0.20	1.56	.260
7--44	47	<0.01	0.38	.941	78	<0.01	0.27	.933

Channel Pairs	OxyHb				DeoxyHb			
	n	*M*	*SD*	2-sided *p*	*n*	*M*	*SD*	2-sided *p*
7--46	47	0.05	0.29	.211	78	<0.01	0.30	.902
18--46	40	0.05	1.36	.812	78	0.08	1.05	.503
19--49	43	0.41	1.86	.151	78	0.19	1.25	.176
20--32†	23	-0.16	0.45	.097	78	0.01	0.29	.857
20--34	42	-0.08	1.57	.737	78	0.04	1.00	.713
21--32	34	-0.04	0.41	.608	78	-0.02	0.40	.703
21--48	52	0.13	0.36	.012*	78	0.08	0.36	.064
23--42	51	0.10	0.75	.356	78	0.07	0.68	.394
24--37	36	0.02	0.34	.703	78	<0.01	0.23	.944
32--47	31	0.04	0.38	.604	78	0.09	0.28	.008*
42--47	**54**	**0.36**	**0.64**	**<.001***	**78**	**0.26**	**0.44**	**<.001***

Note. Descriptive statistics for functional connections between individual channels within each network. Associations that survive Bonferroni corrections ($p \leq .001$) are in bold.

*$p < .05$.

† = Paired samples *t*-test revealed a significant difference ($p < .05$) between mean oxyHb Z-scores and mean deoxyHb Z-scores for the channel pair.

Supplementary Table 2

Correlations Among Study Variables – DeoxyHb Connectivity

Variable	1	2	3	4	5	6	7	8	9	10	11	12	13
1. Household income	–												
2. Maternal education	.575**	–											
3. Maternal sensitivity vs. insensitivity scale	.079	.023	–										
4. Maternal cooperation vs. interference scale	-.030	-.177	.841**	–									
5. FPN deoxyHb connectivity	.190	.340*	-.013	-.010	–								
6. DMN deoxyHb connectivity	.038	-.043	.088	.073	.154	–							
7. HI deoxyHb connectivity	.162	-.108	-.186	-.047	.528**	.282*	–						
8. CN deoxyHb connectivity	-.017	-.114	-.160	-.026	.172	.408**	.419**	–					
9. FC seconds	.010	-.039	-.272	-.176	.023	.146	.107	.076	–				
10. Infant sex assigned at birth	-.161	-.203	.250	.196	-.250	.278*	-.059	-.061	-.023	–			
11. Mother race	-.058	-.027	.213	.126	-.158	-.094	-.326*	-.018	-.038	.040	–		
12. Infant race	-.183	-.101	.079	-.056	-.292*	-.011	-.276	-.109	-.175	.194	.549**	–	
13. Number of children	-.240	-.326*	-.268	-.275	-.268	.022	-.060	.076	.058	.039	-.089	-.021	–

Note. FPN, fronto-parietal network; DMN, default mode network; HI, homologous-interhemispheric connections; CN, control network; FC seconds, number of seconds for which functional connectivity data were available for each participant. Dichotomous variables include infant sex assigned at birth (1 = Male, 2 = Female), and mother and infant race (1 = White, 2 = Not White). $N = 50$.

*$p < .05$, **$p < .01$, two-tailed.

Supplementary Table 3

Correlations Between OxyHb and DeoxyHb Connectivity

Variable	1	2	3	4	5	6	7	8
1. FPN oxyHb	–							
2. FPN deoxyHb	.494**	–						
3. DMN oxyHb	.155	.262	–					
4. DMN deoxyHb	.041	.154	.460**	–				
5. HI oxyHb	.394**	.420**	.276	.242	–			
6. HI deoxyHb	.323*	.528**	.223	.282*	.621**	–		
7. CN oxyHb	.167	.261	.359*	.272	.430**	.149	–	
8. CN deoxyHb	.111	.172	.231	.408**	.369**	.419**	.537**	–

Note. FPN, fronto-parietal network; DMN, default mode network; HI, homologous-interhemispheric connections; CN, control network. $N = 50$.

*$p < .05$, **$p < .01$, two-tailed.
